# Who Was the First Doctor to Report the COVID-19 Outbreak in Wuhan, China?

**DOI:** 10.2967/jnumed.120.247262

**Published:** 2020-06

**Authors:** Xixing Li, Weina Cui, Fuzhen Zhang

**Affiliations:** * The Second Hospital of Hebei Medical University Shijiazhuang, China E-mail: iamcuiweina@126.com

**TO THE EDITOR:** We read with great interest the recent publication entitled “Dr. Li Wenliang and the Time of COVID-19” (1). As mentioned in this article, Dr. Li was reprimanded initially for ‘‘disrupting public order’’ in China when he first reported the outbreak in Wuhan. As a matter of fact, On December 30, 2019, Dr. Li warned in an online chat group on WeChat that he had seen a report showing positive test results of SARS for 7 patients. However, he did not formally report the outbreak to the authorities.

Dr. Zhang Jixian is considered the first doctor to report the novel coronavirus before its outbreak. A senior couple living in the residential community near Dr. Zhang’s hospital went to see her for their fever and cough on December 26, 2019. When she observed their CT thorax images, Dr. Zhang found differences from pneumonia caused by common viruses. Zhang’s experience during the 2003 SARS outbreak, when she worked as a medical expert investigating suspected patients in Wuhan, made her sensitive to signs of an epidemic. After reading the CT images of the elderly couple, she summoned their son, demanding a CT scan of him too. It was Zhang’s insistence that brought her the second piece of evidence: the son’s lungs showed the same abnormalities as those of his parents. Also on December 27, the hospital received another patient who also developed symptoms of coughing and fever and showed the same lung images in the CT scan. The blood tests of the 4 indicated viral infections.

“Usually, a family comes to the hospital and there is little chance for all the family members to have the same disease except for infectious diseases,” said Dr. Zhang, who gave the couple’s family and the patient from the seafood market tests denying the possibility of flu.

As the situation continued to confuse Zhang, she reported it to the head of the hospital on December 27, and the hospital then reported it to the Center for Disease Control (CDC) in the Jianghan district of Wuhan. Back then, knowledge of the virus was scarce. After filing the report, Dr. Zhang cordoned off an area in the department’s ward to hospitalize the 4 patients described above. She then demanded medics in the ward to beef up self-protection. Epidemiologic investigations and tests were arranged quickly by the CDC. Within 2 d, the hospital continued to receive more similar patients, and Dr. Zhang promptly reported to the hospital. Before the CDC expert group paid attention to this epidemic, Dr. Zhang had set up a 9-bed isolation ward for the patients and taken measures to isolate them. She also bought 30 pieces of canvas as protective clothing. “If it is delivered uniformly by the hospital, it needs to be customized” she said, “if it is online shopping, they can receive the goods very quickly.”

At 1:00 pm on December 29, the vice president of the hospital reported directly to the disease control department of the provincial and municipal health and Health Commission. Although December 29 is not a working day, the provincial and municipal health commission disease control department immediately responded to the report and came to the hospital to formally begin the epidemiologic investigation.

At 3:10 pm on December 30, Wuhan Municipal Health and Health Commission issued the official document “emergency notice on reporting the treatment of pneumonia of unknown causes.” The National Health Commission (NHC) dispatched a working group and an expert team in the wee hours of December 31 to Wuhan to guide epidemic response and conduct on-site investigations. Not only was Dr. Zhang the first to sound an alarm for epidemic prevention and control, but she was also the “leader” of hospital rescue work, always putting the interests of the party and people first. By early February, miraculously, none of Dr. Zhang’s team had been infected in Wuhan, where the outbreak was most severe. In February, the Hubei provincial human resources and social security department and the provincial health commission awarded Dr. Zhang for her exemplary service, hailing her as “the first to report the epidemic in the province” and recognizing her leadership and arduous work in the hospital’s fight against COVID-19. However, Dr. Zhang tried to play down the honor and said, “I was just doing what a doctor was supposed to do, driven by professionalism.”

**FIGURE 1.**
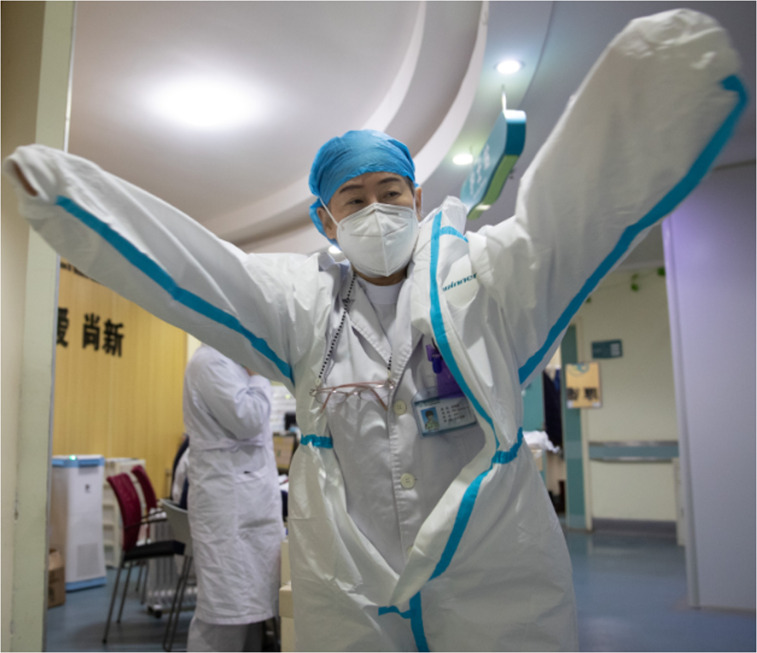
Zhang Jixian, director of the respiratory and critical care medicine department of Hubei Provincial Hospital of Integrated Chinese and Western Medicine, puts on the protective suit before entering the ward at the hospital in Wuhan, central China’s Hubei Province, March 11, 2020.

Therefore, Dr. Zhang was the first to report the novel coronavirus; Dr. Li just blew the whistle. Both of them are worth remembering. At present, Chinese health-care workers have made a miracle in the fight against COVID-19. They have fulfilled their duties with the spirit of patriotism, unity, and dedication. They are considered to be fearless soldiers and heroes.

A salute to health-care workers all over the world!

## DISCLOSURE

No potential conflict of interest relevant to this article was reported.
